# Deregulated NKL Homeobox Genes in B-Cell Lymphoma

**DOI:** 10.3390/cancers11121874

**Published:** 2019-11-26

**Authors:** Stefan Nagel, Hans G. Drexler

**Affiliations:** Leibniz-Institute DSMZ, Department of Human and Animal Cell Lines, 38124 Braunschweig, Germany; hdr@dsmz.de

**Keywords:** homeobox, homeodomain, NKL-code

## Abstract

Recently, we have described physiological expression patterns of NKL homeobox genes in early hematopoiesis and in subsequent lymphopoiesis. We identified nine genes which constitute the so-called NKL-code. Aberrant overexpression of code-members or ectopically activated non-code NKL homeobox genes are described in T-cell leukemia and in T- and B-cell lymphoma, highlighting their oncogenic role in lymphoid malignancies. Here, we introduce the NKL-code in normal hematopoiesis and focus on deregulated NKL homeobox genes in B-cell lymphoma, including *HLX*, *MSX1* and *NKX2-2* in Hodgkin lymphoma; *HLX, NKX2-1* and *NKX6-3* in diffuse large B-cell lymphoma; and *NKX2-3* in splenic marginal zone lymphoma. Thus, the roles of various members of the NKL homeobox gene subclass are considered in normal and pathological hematopoiesis in detail.

## 1. Hematopoiesis and B-Cell Development

**Figure 1:**
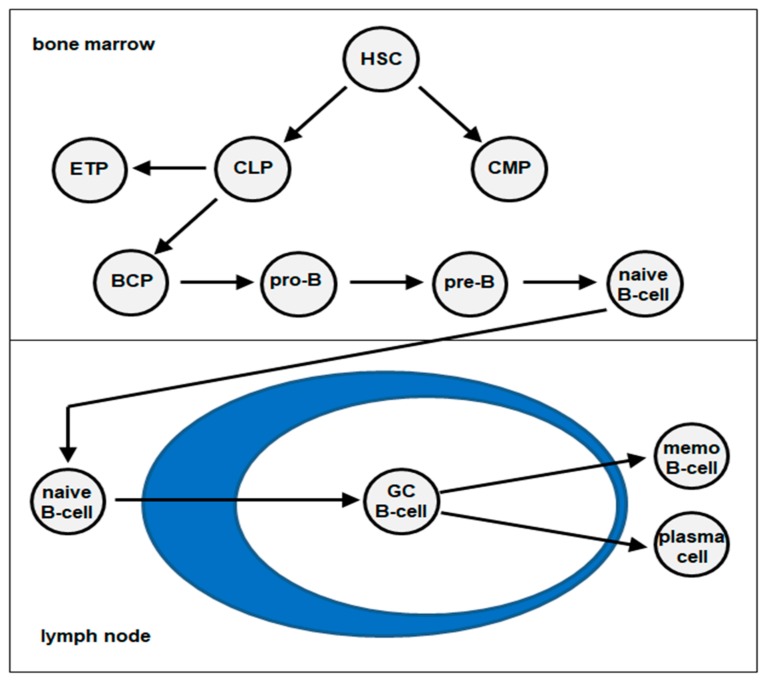
Schematic presentation of B-cell development. Hematopoietic stem cell (HSC), common myeloid progenitors (CMP), common lymphoid progenitors (CLP), early T-cell progenitors (ETP), B-cell progenitor (BCP), germinal center (GC).

The process of hematopoiesis is responsible for the production of all types of blood cells. Hematopoietic stem and progenitor cells (HSCs) generate common myeloid and lymphoid progenitors (CMP and CLP) which, respectively, represent the starting points for the myeloid and lymphoid cell lineages. The latter produces all types of lymphocytes comprising B-cells, T-cells, natural killer (NK)-cells and innate lymphoid cells. Early B-cell development, which includes the rearrangements of the B-cell receptor genes (immunoglobulin heavy chain, *IGH*), takes place in the bone marrow and begins with the CLP-derived B-cell progenitor (BCP). BCPs differentiate via the pro-B-cell and pre-B-cell stages into naïve B-cells. In contrast, early T-cell progenitors (ETP) migrate into the thymus to complete their differentiation. For the final differentiation steps to memory B-cells (memo B-cell) and plasma cells via the stage of germinal center (GC) B-cells, naïve B-cells migrate from the bone marrow into lymph nodes, the spleen and other lymphoid tissues [1,2,3] (Figure 1). In these compartments, additional molecular alterations occur, like somatic hypermutation and class switching of the *IGH* genes. These alterations are performed at the DNA level and the process of class switching is additionally connected with gene rearrangements.

The main regulatory steps of lymphopoiesis including B-cell development are controlled at the transcriptional level [3,4]. Accordingly, several transcription factors (TFs), like BCL6, EBF1, MYB, PAX5, PRDM1 (alias name: BLIMP1) and TCF3 (E2A), are members of a B-cell specific regulatory network which orchestrates basic differentiation processes [5,6,7]. TCF3 plays a prominent role in the development of all types of lymphocytes, while EBF1 and PAX5 are master factors of the B-cell lineage. BCL6 and PRDM1 inhibit each other and are involved in differentiation processes taking place in the GC. Provoked by aberrant chromosomal rearrangements or gene mutations, deregulations of these developmental TFs are thought to contribute to the generation of B-cell malignancies [8,9]. Abnormal rearrangements of the *IGH* genes represent a frequent mechanism of oncogene activation, while deregulated hypermutation is known to be responsible for many gene mutations.

## 2. Classification of Homeobox Genes

Homeobox genes encode TFs, which regulate fundamental processes in development and differentiation in both embryogenesis and the adult. They share the conserved 180 bp long homeobox, which encodes the homeodomain at the protein level. This domain consists of 60 amino acid residues and mediates specific interactions with DNA, chromatin, non-coding (nc)RNA and cooperating TFs, thus representing a common platform of their gene regulatory activities [10].

**Figure 2:**
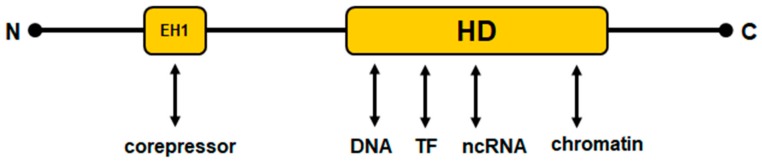
Schematic structure of NKL homeodomain proteins. HD: conserved homeodomain consisting of 60 amino acid residues; EH1: conserved engrailed-homology domain consisting of about eight amino acid residues. The N- and C-terminal parts show no sequence conservation. The EH1 domain and the homeodomain interact with particular components of the gene regulatory machinery.

The subgroup of NK-like homeobox genes, which were later called NKL homeobox genes, have been reported for the first time by Nirenberg and Kim (abbreviated as NK) in the fruit fly *Drosophila*. In this invertebrate organism, the subgroup members are arranged in a cluster consisting of six genes [11]. Additional orthologous genes have since been identified and the group extended. These studies indicated that this clustering represents the ancient gene order, which is now just barely visible in vertebrates [12]. Thus, in contrast to the still-clustered *HOX*-genes, NKL homeobox genes show only relicts of a clustered arrangement in humans. In addition to their conserved homeodomain, NKL homeodomain proteins share a short, conserved sequence in their N-terminal part, which has been termed the engrailed-homology motif (EH1) [13,14]. This motif performs physical interactions with corepressors of the groucho family, thus mediating transcriptional inhibition [15]. Figure 2 depicts a schematic structure of NKL homeodomain proteins. Most NKL homeobox genes are functionally associated with mesodermal development, possibly displaying their ancient function [16]. A systematic classification of all 235 human homeobox genes has generated a panel of eleven classes and several subclasses. Main classes are called antennapedia (ANTP) and paired box (PRD), comprising 150 genes. Other classes identified are CERS, CUT, HNF, LIM, POU, PROS, SINE, TALE, and ZF. Accordingly, NKL homeobox genes represent a subclass of the ANTP class and contain 48 members in humans [17].

## 3. NKL Homeobox Genes in Hematopoiesis

### 3.1. NKL-Code in Developing Lymphocytes

In 2003, we identified the aberrantly expressed homeobox gene *NKX2-5 (CSX1)* in two different T-cell acute lymphoblastic leukemia (T-ALL) derived cell lines, which became activated via the chromosomal rearrangement t(5;14)(q35;q32) [18]. This gene was the third homeobox oncogene identified in this disease after the initial reports of *TLX1 (HOX11)* and *TLX3 (HOX11L2)* in 1991 and 2001, respectively [19,20,21]. We recognized that all three genes are members of the same group of NKL homeobox genes and suggested that these related genes may thus perform similar oncogenic effects [18]. To date, 24 aberrantly activated NKL homeobox genes have been described in T-ALL patients, representing the largest group of oncogenes in this malignancy [22,23]. These oncogenes additionally include *MSX1, NKX2-1, NKX3-1*, and *NKX3-2* [24,25,26,27,28,29]. Mechanisms of aberrant gene activation are presented by chromosomal rearrangements and deregulated activites of TFs, chromatin factors, and signalling pathways [18,24,27,30]. Furthermore, deregulated NKL homeobox genes play a significant role in T-cell lymphoma as well, underlining their oncogenic potential in T-cells [31].

**Figure 3:**
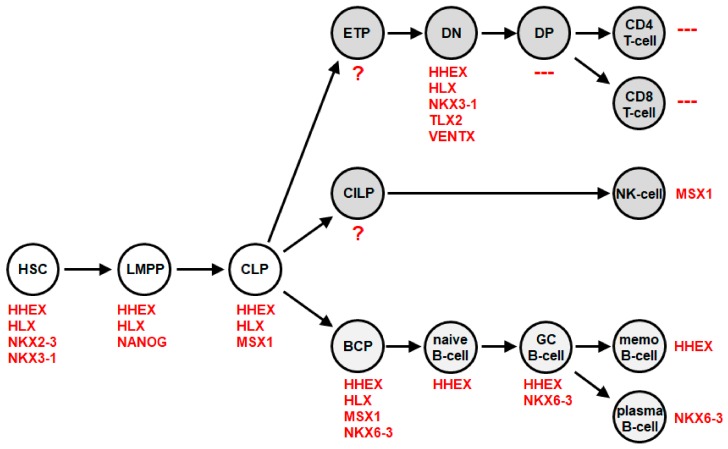
The NKL-code in lymphopoiesis This diagram depicts activities of NKL homeobox genes during early hematopoiesis and lymphopoiesis, including the development of T-cells, NK-cells and B-cells. Each cell/stage is labelled with the accordingly expressed NKL homeobox genes. BCP: B-cell progenitor, CILP: common innate lymphoid progenitor, CLP: common lymphoid progenitor, DN: double negative, DP: double positive, ETP: early T-cell progenitor, GC B-cell: germinal center B-cell, HSC: hematopoietic stem cell, LMPP: lymphoid primed multipotent progenitor, memo B-cell: memory B-cell.

Then, we analyzed the physiological activity of NKL homeobox genes in early hematopoiesis and T-cell development. This exercise revealed nine members, comprising *HHEX, HLX, MSX1, NANOG, NKX2-3, NKX3-1, NKX6-3, TLX2,* and *VENTX (*Figure 3). They showed a specific expression pattern in stem cells, progenitor cells and immature thymocytes, but not in mature T-cells, which tested negative. We named this pattern and the respective genes the NKL-code [22]. This code demonstrated that most NKL homeobox oncogenes in T-ALL are ectopically expressed. For example, *NKX2-5* is normally expressed in the developing heart and spleen but not in any hematopoietic cell [32]. Furthermore, *MSX1* is normally expressed in hematopoietic progenitors, including CLP and BCP in addition to mature NK-cells, but not in the T-cell lineage (Figure 3). Accordingly, *MSX1* is an oncogene in T-ALL and a tumor suppressor in NK-cell leukemia [24,33,34]. *HLX (HLX1, H2.0* or *HB241*) was the first described NKL homeobox gene that is physiologically expressed in hematopoietic cells, including myeloid and B-cells, but not in T-cells [35]. These data support the reported expression pattern of NKL-code members and highlight their functional role in leukemia.

Additional gene codes containing NKL homeobox genes have been published for the developing neural tube, pharyngeal region, and teeth [36,37,38]. The code members for the neural tube comprise *DBX1, DBX2, NKX2-2, NKX6-1*, and *NKX6-2* [36]. In the pharyngeal region, the gene code consists exclusively of all six DLX family members, while in developing teeth, *BARX1, DLX1, DLX2, MSX1*, and *MSX2* create a code [37,38]. Most of those NKL homeobox gene code members are regulated by signalling pathways and perform cross-reactivity. In the neural tube, the hedgehog- and BMP-pathways are regulated by ligand gradients which are created in opposite directions, thus regulating NKL homeobox gene activities [36]. Therefore, differentiation processes are frequently controlled by particular NKL homeobox genes, via formation of a code.

### 3.2. B-Cell Associated NKL Homeobox Genes In Normal Development

In 2018, we reported an extended version of the NKL-code, which included developing and mature B-cells [39]. This study revealed four NKL homeobox genes expressed in the B-cell lineage, namely *HHEX, HLX, MSX1*, and *NKX6-3* (Figure 3). BCPs express *HHEX, HLX, MSX1*, and *NKX6-3*, while mature memory B-cells express just *HHEX,* and mature plasma cells *NKX6-3*. Thus, at each stage of physiological B-cell differentiation, particular NKL homeobox genes are active, generating a specific pattern. Moreover, as described for other NKL homeobox gene codes, hematopoietic NKL-code members show cross-reactivity as well: *HHEX* and *HLX* repress *MSX1* and *NKX6-3; NKX6-3* represses *MSX1*; and *MSX1* activates *NKX6-3* [39].

Two of these genes, *HLX* and *HHEX* (*HEX* or *PRH*), represent the first described non-*HOX* homeobox genes expressed in hematopoietic cells [35,40]. Expression analyses of these two genes revealed activity in B-cells and myeloid cells, while T-cells were described to be negative [35,41,42,43]. Moreover, downregulation of *HHEX* was shown to be crucial for normal T-cell differentiation [44]. Of note, the data failed to detect *HHEX* in plasma cells, consistent with our screening data for the NKL-code [39,43]. Accordingly, analysis of *HHEX*-knockout mice showed reduced numbers of both mature and pre-B-cells, demonstrating the importance of *HHEX* for B-cell development [45,46]. Forced expression of *HLX* in hematopoietic progenitors enhanced myeloid differentiation but arrested the development of B-cells at the pro-B-cell stage and of T-cells at the CD4/CD8 double-positive stage [41,47]. In T_H_1-cells, *HLX* is induced by the TF TBX21 (TBET) and thereby involved in the expression of interferon gamma *IFNG* [48,49]. However, in NK-cells, HLX performs negative regulation of *IFNG*, demonstrating context-dependent control of target genes [50]. Collectively, these data highlight *HHEX* and *HLX* as important regulators of hematopoiesis, including B-cell differentiation.

*HHEX*: In addition to their activity in developing B-cells, the four NKL-code members *HHEX, HLX, MSX1,* and *NKX6-3* are involved in the differentiation of other tissues and organs as well. *HHEX* is expressed in parts of the early embryo, including the chorion and yolk sac, later in primordia of the liver and the thyroid, and then in fetal liver, thyroid and lung [51,52]. *HHEX* is also expressed in the 3rd pharyngeal pouch, which generates the thymus, and in primordia of the pancreas and the gallbladder [53]. Furthermore, *HHEX* plays a role in vascular and lymphatic development [54]. The expression of *HHEX* was also detected in normal breast tissue [55]. Accordingly, *HHEX* performs tumor suppressor activities in breast cancer [56]. In the embryonal anterior endoderm which generates the lung, thyroid, pancreas, and the liver, *HHEX* is regulated by a complex network which includes the BMP- and WNT-signalling pathways and the TFs LIM1, NODAL, OTX2, and VENTX [57]. Components of this network may play a regulatory role in the hematopoietic system, as well.

*HLX*: The embryonal expression pattern of *HLX* indicates some degree of overlap with *HHEX*, as shown in the foregut, liver, gallbladder, and lung. In addition, the splanchnic mesoderm and mesenteric tissues tested positive for *HLX* activity in the embryo [58]. *HLX* is also expressed in the placenta, and controls the switch from white to brown adipose tissue [59,60]. Furthermore, *HLX* is involved in the differentiation of embryonal stem cells into the intestinal lineage [61]. A screening in induced pluripotent stem cells (iPSCs) for genes regulating pluripotency revealed *HHEX* and *HLX* [62]. Thus, both genes are involved in pluripotency and reprogramming, highlighting their potential in cell differentiation processes [62].

*MSX1*: In early embryogenesis, *MSX1* is expressed in the neural plate border region and is thus involved in the generation of neural crest cells and the preplacodal ectoderm [63,64,65,66]. These cells generate a multiplicity of tissues and structures which are fundamental for vertebrate development and evolution. Later on, *MSX1* is expressed in neural crest cells and their derived tissues, including the teeth [67,68]. *MSX1* also plays a role in the development of craniofacial structures and neural tissues, including the brain [69,70,71]. Accordingly, mutations in the *MSX1* gene are frequently found to be connected to particular malformations of craniofacial tissues. Furthermore, *MSX1* is expressed in the mammary gland [72]. Functionally, *MSX1* is able to dedifferentiate cells. In muscle cells, it has been shown that *MSX1* dedifferentiates myotubes by repressing the muscle master factor MYOD and the muscle-specific myogenin [73,74]. These data compellingly demonstrated the developmental potential of this TF.

*NKX6-3*: Finally, *NKX6-3* is expressed in the developing stomach and hindbrain [75,76,77]. Depleting mutations of *NKX6-3* in gastric epithelial cells result in the activation of APOBEC family members, which, in turn, enhance the generation of additional mutations and, subsequently, gastric cancer [76]. In addition to *MSX1, NKX6-3* is expressed in the neural plate border region as well. Knockdown and overexpression experiments indicated a dominant role of *NKX6-3* in the development of neural crest cells [78]. *NKX6-3* was able to induce an ectopically neural crest when overexpressed, while *NKX6-3* knockdown generated defects in the neural crest [78]. Taken together, experimental data of *MSX1* and *NKX6-3* highlight the importance and impact of NKL homeobox genes for these pluripotent cells.

Regulation of different developmental processes by the same pathways and TFs, including homeodomain proteins, is a frequent observation in embryogenesis [79]. For example, NKL homeobox gene *NKX2-5* basically regulates the development of both heart and spleen [32,80,81]. Therefore, aberrant activities of developmental homeodomain TFs may recapitulate these tissue-specific operations ectopically or at the wrong stage of differentiation, which may lead to tumorigenesis. NKL homeobox gene *NKX3-1* performs master gene activities in the development of the prostate and plays a role in the early stages of hematopoiesis, showing physiological activity in different tissues [22,28]. NKL homeobox gene *NKX2-5* represents a master gene for the development of the heart in vertebrates and thereby regulates the expression of specific target genes, including *MEF2C* [80]. This function is reactivated in T-ALL and mediates the ectopic deregulation of *MEF2C,* which constitutes a major oncogene in this malignancy [18,25,82,83]. Thus, consideration of known physiological operations of aberrantly activated NKL-code members and non-members may reveal findings of clinical importance. The druggability of NKL homeobox genes in particular, and of TFs in general, is difficult to define, but their regulated genes may represent suitable and effective targets for therapy [84].

### 3.3. Deregulated NKL Homeobox Genes In B-Cell Malignancies

Developmental arrest is a main and widespread feature of cancer cells [85,86,87]. As shown by Allen and coworkers in 1995, forced expression of HLX in hematopoietic progenitors resulted in developmental arrest of pro-B-cells [47]. Therefore, this experiment represents the first hint of the oncogenic potential of NKL homeobox genes in B-cells. Ferrando and coworkers correlated aberrant expression of NKL homeobox gene *TLX1* with the developmental arrest of malignant thymocytes at the double-positive stage in T-ALL [88]. Thus, the correlation of aberrantly expressed NKL homeobox genes with particular stages of differentiation from lymphocytes highlights the developmental impact of this gene subclass in lymphoid tumors. Recently, we screened deregulated NKL homeobox genes in a variety of B-cell malignancies, which included Hodgkin lymphoma (HL), follicular lymphoma (FL), diffuse large B-cell lymphoma (DLBCL), hairy cell leukemia (HCL), mantle cell lymphoma (MCL), and splenic marginal zone lymphoma (SMZL). This study revealed 13 aberrantly overexpressed NKL homeobox genes (Table 1), supporting the relevance of this gene group for B-cell cancers [39]. In the following, we discuss in more detail seven selected NKL homeobox genes which have been studied by us and others in B-cell lymphomas, both in primary cells and in cell lines.

*HLX*: *HLX* is a member of the hematopoietic NKL-code and is expressed in early hematopoiesis and BCPs [22,39]. Aberrant expression of *HLX* has been found in HL, FL, MCL, and SMZL [39]. *HLX* is part of a regulatory network consisting of B-cell associated NKL-code members *HHEX, MSX1* and *NKX6-3* [39]. Aberrant overexpression of *HLX* in HL cell line L-540 mediated downregulation of *MSX1* and *NKX6-3*, in addition to B-cell factors BCL11A and SPIB, and of pro-apoptotic factor BCL2L11 (BIM) [89]. These regulatory relationships may underlie the described differentiation arrest in immature B-cells after forced expression of *HLX* [47]. Furthermore, they enhance survival of the tumor cells which represent a main property of HL [89]. ChIP-seq data from ENCODE indicated *HLX* as target gene of STAT3, which is a transcriptional mediator of several signalling pathways [90]. Accordingly, aberrant activation of STAT3 by JAK2-mediated phosphorylation and/or HDAC-mediated deacetylation contributes to enhanced *HLX* expression in HL [89]. Acetylation of STAT3 was shown to regulate its nuclear localization, representing an additional level of gene regulation which depends on the activity of acetylating and deacetylating enzymes. Of note, HDACs are druggable enzymes and, thus, potential targets for rational therapies to inhibit STAT3-signalling. Taken together, *HLX* is embedded in major oncogenic disturbances of HL, including aberrant signalling and apoptosis.

*MSX1: MSX1* is normally expressed in CLPs and BCPs and downregulated in the course of B-cell development [39]. Aberrantly overexpressed *MSX1* in HL cell line L-1236 inhibited the expression of the B-cell factor ZHX2, probably using histone H1 as co-repressor [91,92]. Silencing of ZHX2 may contribute to the deregulated B-cell phenotype in HL. In ovarian cancer cells, *MSX1* performs inhibition of cyclin D1 (*CCND1*) in addition to other cyclins and cell cycle regulators, while, in primary mesenchymal and epithelial progenitor cell types, *MSX1* activates *CCND1* [93,94]. These data reflect a balanced interplay between proliferation and differentiation. Accordingly, an inhibitory impact of *MSX1* on *CCND1* expression was lost in MCL cells containing chromosomal translocation t(11;14)(q13;q32). This genomic rearrangement separates the regulatory *MSX1* binding site from the coding part of *CCND1* [95]. Therefore, this oncogenic alteration disturbs the indicated balanced interplay and may support CCND1-mediated cell proliferation in MCL.

*NKX2-1: NKX2-1* (TTF1) expression has been identified in the DLBCL cell line SU-DHL-5, representing the first documentation of an aberrantly activated NKL homeobox gene in B-cell malignancies [30]. Patient data indicated *NKX2-1* deregulation in about 5% of DLBCL cases [30,39]. While, in T-ALL, a chromosomal translocation aberrantly activated *NKX2-1* in SU-DHL-5 cells, TF HEY1 and chromatin factors KMT2A (MLL,) and particular histones, are involved in *NKX2-1* deregulation [25,30]. KMT2A overexpression in this cell line was correlated with a chromosomal duplication at 11q23 and overexpression of histone H2B, with a chromosomal abnormality at 6p22 [30]. Normally, *NKX2-1* is expressed in the embryonal thyroid, lung and brain but not in hematopoietic cells and tissues at any time [96]. Thus, *NKX2-1* is ectopically activated in different lymphoid malignancies.

*NKX2-2:* Similar to *NKX2-1, NKX2-2* is ectopically activated in lymphoid tumors, including T-ALL and HL [22,25,97]. In both classical and nodular lymphocyte predominant HL, about 12% of the patients express *NKX2-2* [97]. *NKX2-2*-expressing HL cell line DEV served as a model to reveal aberrant mechanisms of activation. Normally, *NKX2-2* is expressed in the brain and pancreas [98,99]. Accordingly, aberrant reactivation of neural pathways and TFs, including IL17RB, FOXG1, FLI1 and NEUROD1, were found to be responsible for *NKX2-2* expression in cell line DEV [97]. Of note, *IL17RB* is targeted by chromosomal translocation t(3;14)(p21;q32). Furthermore, IL17RB activator DAZAP2 is overexpressed via a chromosomal duplication at 12p13, while the gene encoding its repressor, *SMURF2*, is deleted at 17q24. Thus, three different chromosomal aberrations contribute to activated IL17RB-signalling and subsequent *NKX2-2* transcription [97]. Therefore, *NKX2-2* expression in HL exemplifies that ectopic reactivation of particular developmental pathways and factors may result in aberrant expression of NKL homeobox genes in B-cell malignancies.

*NKX2-3: NKX2-3* is normally expressed in HSCs, thus representing a hematopoietic stem cell factor [22]. In addition, *NKX2-3* acts as a master gene for the embryonal development of the spleen [100]. Thus, NKX2-3 is closely associated with the differentiation of hematopoietic cells and tissues. However, in advanced hematopoietic stages or mature blood cells *NKX2-3* is silenced. In SMZL, *NKX2-3* is aberrantly activated by chromosomal translocation t(10;14)(q24;q32), juxtaposing the locus of this NKL homeobox gene to that of *IGH* [101]. Downstream analyses indicated aberrant activation of B-cell receptor signalling, enhanced expression of integrins, adhesion factor MADCAM1, and of chemokine receptor CXCR4 [101]. These features may underlie malignant transformation and homing of the tumor cells to the spleen and lymph nodes. In addition to SMZL, aberrant expression of *NKX2-3* has been detected in DLBCL, FL, MCL, chronic lymphoid leukemia, and multiple myeloma (MM) [101]. Furthermore, deregulated expression of *NKX2-3* has been associated with Crohn disease, ulcerative colitis and inflammatory bowel disease [102]. These diseases originate from immunological disturbances which may share certain pathological aspects with particular B-cell malignancies.

*NKX6-3: NKX6-3* is a member of the NKL-code and normally expressed in BCPs, GC B-cells and plasma cells [39]. This pattern indicates important functions for the differentiation of B-cells and the status of plasma cells. Aberrant overexpression of *NKX6-3* was detected in FL, DLBCL and MCL patients [39]. DLBCL cell line DOHH-2 showed overexpression of *NKX6-3* that was mediated by aberrant BMP-signaling and enhanced activity of chromatin factor AUTS2 [39]. Interestingly, infection of this cell line with Epstein–Barr virus (EBV) resulted in enhanced expression of *HLX* via STAT3 which in turn repressed *NKX6-3* [103]. The EBV-encoded factors LMP1 and LMP2A were shown to mediate STAT3 activation in this cell line. These data may explain the described malignant associations of EBV and B-cell lymphomas [104].

*BARX2: BARX2* is normally expressed during embryogenesis in several tissues, including the nervous system, Rathke´s pouch and submandibular glands, and in the adult ovarian epithel [105,106,107]. However, *BARX2* is not expressed in developing or adult hematopoietic cells or tissues. Aberrant activity of *BARX2* has been found in HCL and MCL patients [39]. Furthermore, *BARX2* and *NKX2-3* were the only NKL homeobox genes aberrantly expressed in MM [101,108]. Interestingly, except for *BARX2*, MM and primary effusion lymphoma cell lines lacked activity of any NKL-code member, indicating aberrant downregulation of *HHEX* and *NKX6-3.* Thus, in mature B-cell malignancies, NKL homeobox genes may perform tumor suppressor activity.

### 3.4. Cell Lines as Models for Deregulated NKL Homeobox Genes

Cell lines represent experimental models for that tumor type from which they were derived. Of note, it is of fundamental importance to use authenticated, well characterized, and annotated cell lines to be able to extrapolate cell line data to particular cancers, including B-cell malignancies. We have evaluated and systematically listed hematopoietic cell lines which meet these criteria [109]. In addition, we reviewed cell lines derived from several entities of B-cell malignancies, including B-cell precursor-leukemia, MM, primary effusion lymphoma, primary mediastinal B-cell lymphoma, double-hit B-cell lymphoma, and HL, to highlight appropriate models for particular tumors [110,111,112,113,114,115]. Here, we presented data obtained from both patients and cell lines. Deregulated NKL homeobox genes were usually identified in patients and subsequently investigated in cell lines, analyzing mechanisms of deregulation and downstream activities. Table 2 shows malignant B-cell lines and corresponding aberrant NKL homeobox gene activities to help identify models which would be suitable for the type of cancer and/or the gene of interest.

## 4. Conclusions

NKL homeobox genes are physiologically expressed in hematopoiesis including B-cell development, in a specific pattern which we have termed the NKL-code. Aberrant activities of these basic developmental regulators are involved in the pathogenesis of B-cell malignancies. The knowledge of their pathophysiological activity and the understanding of their function may contribute to improved diagnostics and novel therapies in the future. Finally, in this field of research, validated cell lines represent informative models to explore the landscape of NKL homeobox genes.

## Figures and Tables

**Table 1:** NKL homeobox gene expression in normal B-cell development and B-cell lymphomas.

Gene	HSC	LMPP	CLP	DN	DP	BCP	NB	GCB	MB	PC	HL	FL	DLBCL	HCL	MCL	SMZL
**HHEX**																
**HLX**																
**MSX1**																
**NANOG**																
**NKX2-3**																
**NKX3-1**																
**NKX6-3**																
**TLX2**																
**VENTX**																
**BARX2**																
**DLX1**																
**EMX2**																
**NKX2-1**																
**NKX2-2**																
**NKX3-2**																

Physiologically expressed genes are indicated in red, deregulated gene activities detected in patients are indicated in orange. BCP: B-cell progenitor, CLP: common lymphoid progenitor, DLBCL: diffuse large B-cell lymphoma, DN: double negative, DP: double positive, FL: follicular lymphoma, GCB: germinal center B-cell, HCL: hairy cell leukemia, HL: Hodgkin lymphoma, HSC: hematopoietic stem cell, LMPP: lymphoid primed multipotent progenitor, MB: memory B-cell, NB: naive B-cell, PC: plasma cell, MCL: mantle cell lymphoma, SMZL: splenic marginal zone lymphoma. This table is modified as described previously [39].

**Table 2:** Aberrantly expressed NKL homeobox genes in malignant B-cell lines.

Gene	Cell Line	Disease	Remarks	Reference
***HHEX***				
***HLX***	L-540 DOHH-2 OCI-LY19 NU-DHL-1 SEM	HL DLBCL DLBCL DLBCL BCP-ALL	elevated STAT3 activity EBV-mediated STAT3 activation	[79] [35,94] [99] [99] [99]
***MSX1***	L-1236 GRANTA-519 JEKO-1 REC-1	HL MCL MCL MCL	cofactor H1C, target *ZHX2* t(11;14)(q13;q32) activates *CCND1* t(11;14)(q13;q32) activates *CCND1* t(11;14)(q13;q32) activates *CCND1*	[82] [85] [85] [85]
***NANOG***				
***NKX2-3***				
***NKX3-1***				
***NKX6-3***	DOHH-2	DLBCL	repressed by HLX	[35,94]
***TLX2***				
***VENTX***	SEM	BCP-ALL		[99]
				
***BARX2***	RPMI-8226	MM		[99]
***DLX1***				
***EMX2***				
***NKX2-1***	SU-DHL-5	DLBCL	KMT2A, H2B	[86]
***NKX2-2***	DEV	HL	activated by IL17RB-signalling	[88]
***NKX3-2***				

This table lists B-cell lines in which particular NKL homeobox genes are overexpressed. Additionally, the corresponding disease and potentially relevant information are given. The abbreviations are explained in Table 1.
